# Psychometric Evaluation of the Revised Michigan Diabetes Knowledge Test (V.2016) in Arabic: Translation and Validation

**DOI:** 10.1155/2016/9643714

**Published:** 2016-11-23

**Authors:** Ali Hassan Alhaiti, Alanod Raffa Alotaibi, Linda Katherine Jones, Cliff DaCosta, George Binh Lenon

**Affiliations:** ^1^School of Health and Biomedical Sciences, RMIT University, Bundoora West Campus, Bundoora, VIC 3083, Australia; ^2^Nursing Education Department, King Fahad Medical City, Riyadh, Saudi Arabia; ^3^Specialized Diabetes and Endocrine Centre, King Fahad Medical City, Riyadh, Saudi Arabia; ^4^School of Science, RMIT University, Bundoora West Campus, Bundoora, VIC 3083, Australia

## Abstract

*Objective*. To translate the revised Michigan Diabetes Knowledge Test into the Arabic language and examine its psychometric properties.* Setting*. Of the 139 participants recruited through King Fahad Medical City in Riyadh, Saudi Arabia, 34 agreed to the second-round sample for retesting purposes.* Methods*. The translation process followed the World Health Organization's guidelines for the translation and adaptation of instruments. All translations were examined for their validity and reliability.* Results*. The translation process revealed excellent results throughout all stages. The Arabic version received 0.75 for internal consistency via Cronbach's alpha test and excellent outcomes in terms of the test-retest reliability of the instrument with a mean of 0.90 infraclass correlation coefficient. It also received positive content validity index scores. The item-level content validity index for all instrument scales fell between 0.83 and 1 with a mean scale-level index of 0.96.* Conclusion*. The Arabic version is proven to be a reliable and valid measure of patient's knowledge that is ready to be used in clinical practices.

## 1. Introduction 

Diabetes mellitus, as defined by the World Health Organization [1], is “a metabolic disorder of multiple aetiology characterized by chronic hyperglycaemia with disturbances of carbohydrate, fat and protein metabolism resulting from defects in insulin secretion, insulin action or both.” The International Diabetes Federation (IDF) has indicated that there are 415 million people with diabetes in the world and this number is expected to rise to 642 million by 2040. Conversely, only 12% of global health expenditure, estimated at a cost of US $673 billion, is directed toward diabetes [2].

Saudi Arabia, according to data released by IDF, is one of the top five countries for the prevalence of diabetes in the middle-eastern and north-African regions, with 3.8 million patients with diabetes, which represents 23.9% of the population. This may be due to different cultural structures, active socioeconomic growth, and significant recent adjustments in lifestyle [2, 3]. Studies reveal that diabetes cost Saudi Arabia an estimated $9.4 billion in 2010, a figure that has been predicted to increase sevenfold to 6.5 billion by 2020 [3].

Diabetes self-management education (DSME) is a critical approach that provides the foundation to assist people with diabetes to make a multitude of conventional self-management arrangements and to perform multiple care activities. The primary intention of DSME is to encourage active self-care behaviour facilitating the knowledge, skill, and ability required for diabetes self-care [4]. Further, studies have reported that the utilisation of healthcare services and facilities decreases when people receive educational support compared with those who do not. DSME helps control HbA1c levels an effect that likewise results in significant healthcare savings [5].

The estimation of patient's levels of knowledge has been the cornerstone of medical assessment for many years. In 1998, the diabetes knowledge test (DKT) was validated and introduced as a reliable instrument for the expert evaluation of patients' general knowledge of diabetes [6]. Since then, the test has been used by diabetes researchers throughout the world and translated into multiple languages, such as Spanish, Greek, Navajo, Norwegian, Arabic, and Malaysian [7].

The DKT is a 23-item instrument designed to assess patient knowledge of diabetes concerning diet, exercise, blood glucose levels, and testing and self-care activities. The first 14 items apply to all patients and the remaining nine items are relevant to those using insulin [8]. A 2011 review of the 1998 version of the DKT showed that the test was outdated and needed to be updated according to evidence provided by more recent literature [6]. The DKT has since been revised and modified based on current self-management education and practice standards and was renamed DKT-2 in 2016. No items were added to or withdrawn from the new DKT-2 and most modifications were minor. Seven items were adjusted to simplify the questions and answers, two items were modified to improve the grammar, and the last four items were changed to meet current national standards [6].

The aim of this current study is to translate a valid version of the DKT-2 into Arabic and to evaluate the psychometric properties of this new Arabic version.

## 2. Methods

**Figure 1:**
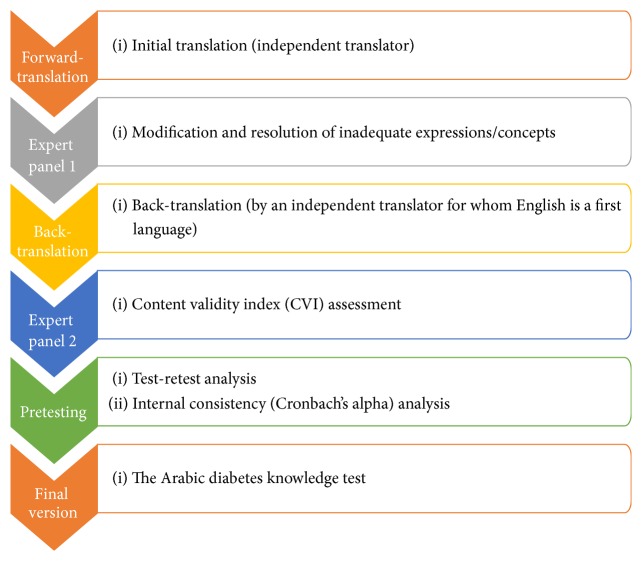
Flowchart depicting the processes used for the translation and validation of the instrument.

The translation of the DKT-2 was conducted in accordance with the WHO process for the translation and adaptation of instruments [1]. Professional translation and validation of texts are attained through several distinct steps, as shown in Figure 1: forward- and back-translation, expert panel review, pretesting, cognitive interviewing, and psychometric evaluation.

### 2.1. Study Setting and Sampling Procedures

This study was conducted in the Specialised Diabetes and Endocrine Centre in King Fahad Medical City (KFMC), which is one of the largest health care facilities in the Gulf region with a total capacity of 1059 beds. The centre consists of four hospitals and multiple departments providing tertiary care for all patients across the Kingdom of Saudi Arabia.

A convenience sample of 139 participants was identified between September and October 2015. Participants were required to meet the following inclusion criteria at the time of the study in order to be considered: (1) they were patients at KFMC, (2) they were at least 20 years old, (3) they had been diagnosed with type 2 diabetes (T2D), and (4) they were able to read and write in Arabic or English.

### 2.2. Procedure

After providing informed consent, the recruited participants were asked to complete a questionnaire. The haemoglobin A1c (HbA1c) levels were obtained from medical records with permission of patients. A researcher was available to answer any questions arising from the questionnaire. To determine test-retest reliability, participants were informed that there would be a follow-up appointment in two weeks' time. In total, 34 participants completed the questionnaire twice.

### 2.3. Ethical Considerations

This study received ethics approval from the ethics committee at KFMC in Saudi Arabia (H-01-R-012), IRB with OHRP/NIH, USA (IRB00008644), and RMIT University in Australia (ASEHAPP 59–14).

### 2.4. Process of Translation and Validation

#### 2.4.1. Forward-Translation

In the first stage, the DKT-2 was translated into Arabic for a fee by a professional independent translator. The translator was requested to retain the concepts, to use appropriate language to reach the broadest possible audience, and to comply with the WHO general guidelines [1]. The assignment was completed over five days and, with the return of the forward-translation, the first Arabic version was ready.

#### 2.4.2. Expert Panel 1

Researchers organized a panel group, consisting of five members including the original translator, experts in health, and experts experienced in research-instrument adoption, according to WHO recommendations. The panel reviewed all related materials provided by the principal investigators along with the translation and were requested to identify and modify any inadequate expressions or concepts. Their recommendations were adopted for the DKT-2, including the phrases utilised in items one, two, three, four, and eight for non-US-patient populations.

The expert panel discussed the individual words and expressions comprising each item and suggested alternatives. Each of the recommendations made by the panel members was considered, except for those that the majority of the panel were able to clearly justify dismissing. On the completion of this stage, the translated text was ready for back-translation.

#### 2.4.3. Back-Translation

The DKT-2 was sent to a second independent and this time native-English-speaking translator, who had not yet engaged in this process and who had no prior knowledge of the study, for back-translation into English. The job was completed and sent back to the researcher three days later. The back-translated version was remarkably similar to the original text with the exception of the sections recommended for non-US-patient populations.

#### 2.4.4. Expert Panel 2

For the second round of revision and modification, the researcher invited another bilingual panel group to assess the content validity index (CVI) and to prepare the final version of the Arabic DKT-2. As shown in Table 1, committee members of both sexes were recruited with the majority of these experts being nurses; however, the sample also included a clinical educator, a clinical-research consultant, the head of diabetes education, and a translator. All worked at KFMC in Riyadh.

Content validity has been defined as “the degree to which an instrument has an appropriate sample of items for the construct being measured” [9]. CVI is the most broadly utilised index in content assessment. It consists of two characters: the item-level content validity index (I-CVI) defining the content validity of individual items and the scale-level content validity index (S-CVI) determining the content validity of an overall scale [10]. Researchers confirm that an acceptable content validity has I-CVI of 0.78 or higher and proportionate unanimous approval or S-CVI/universal agreement (S-CVI/UA) of 0.8 or 0.9 or greater [11].

Studies recommend that a minimum of three experts should engage in this task and that a 4-point scale should be employed to rate the items, with 1 = not relevant, 2 = somewhat relevant, 3 = quite relevant, and 4 = highly relevant [11]. The purpose of the use of CVI is to determine the cultural appropriateness and effectiveness of the DKT-2 Arabic version in measuring patient levels of knowledge in the Arabic-speaking population.

### 2.5. Statistical Analysis

All statistical calculations were undertaken using the Statistical Package for the Social Sciences (SPSS v.23) software. Descriptive statistics were used to describe participant characteristics. Test-retest reliability (*n* = 34) was determined by the intraclass correlation coefficient (ICC) using a two-way, random form. ICC of 0.75 is considered an excellent level of test-retest reliability, rates between 0.40 and 0.75 reflect a good level of reliability, and rates of less than 0.4 indicate poor test-retest reliability [12]. Cronbach's alpha was used to evaluate the text's internal consistency; an acceptable Cronbach alpha score for internal consistency is 0.70 and above [13].

## 3. Results

### 3.1. Participant Characteristics

In total, 139 patients participated in the first round but only 34 (or 24%) were admitted to the second round. Demographic data reflected HbA1c level, age, gender, monthly income, level of education, time since T2D diagnoses, and number of diseases contracted. As shown in Table 2, 51% (*n* = 71) of participants indicated poor levels of HbA1c-level control, 40.3% (*n* = 56) were between 18 and 30 years of age, 44.6% (*n* = 62) were males and 53.2% (*n* = 74) were females, 40.3% (*n* = 56) received less than SR$5000 per month, 41% (*n* = 57) held a bachelor degree, 44% (*n* = 61) had been diagnosed with T2D for more than 10 years, and 38% (*n* = 53) had more than one disease.

### 3.2. Reliability and Validity of the Arabic DKT-2

The Arabic DKT-2 received an internal consistency score of 0.75, which is within the recommended range of Cronbach's alpha test [7]. The outcomes of the test-retest (see Table 3) revealed excellent instrument reliability with a mean ICC of 0.90 [12]. The content validity analysis presented in Table 4 depicts the I-CVI for all instrument scales as between 0.83 and 1 and a mean S-CVI of 96, indicating strong agreement between the two versions. According to Lynn [14], the I-CVI should be no lower than 0.78 or 0.80 for the S-CVI to be judged acceptable.

## 4. Discussion and Conclusion

This study sought to evaluate the translation of the DKT-2, the most commonly used instrument for determining knowledge of diabetes care and management, from English into Arabic [7]. The results demonstrated that the Arabic DKT-2 questionnaire is an acceptable cross-cultural research instrument as shown in the following list which could be used in Saudi Arabia. It was a challenge to translate this text into another language taking into consideration cultural differences [15, 16]. The translation and validation process followed the recommendations of WHO for the translation and adaptation of instruments [1], which included forward-translation conducted by an independent translator followed by a systematic panel meeting to discuss the translation's quality and modify the instrument in keeping with WHO's guidelines. The next step required another independent translator to perform a back-translation before a third version of the Arabic DKT-2 was produced and, finally, another panel met to discuss the CVI and translation outcomes. This panel reported that the I-CVI for all instruments was between 0.83 and 1, with a mean S-CVI of 96, indicating excellent agreement according to Lynn [14]. Statistical psychometric analyses determined that the DKT-2 received the acceptable result of 0.75 for its internal consistency (Cronbach's alpha test) as well as an excellent reliability ICC level of 0.90.

*Michigan Diabetes Research and Training Center's Revised Diabetes Knowledge Test*

1. The diabetes diet is:
  - The way most American people eat
  - A healthy diet for most people
  - Too high in carbohydrate for most people
  - Too high in protein for most people
2. Which of the following is highest in carbohydrate?
  - Baked chicken
  - Swiss cheese
  - Baked potato
  - Peanut butter
3. Which of the following is highest in fat?
  - Low fat (2%) milk
  - Orange juice
  - Corn
  - Honey
4. Which of the following is a “free food”?
  - Any unsweetened food
  - Any food that has “fat free” on the label
  - Any food that has “sugar free” on the label
  - Any food that has less than 20 calories per serving
5. A1C is a measure of your average blood glucose level for the past:
  - Day
  - Week
  - 6–12 weeks
  - 6 months
6. Which is the best method for home glucose testing?
  - Urine testing
  - Blood testing
  - Both are equally good
7. What effect does unsweetened fruit juice have on blood glucose
  - Lowers it
  - Raises it
  - Has no effect
8. Which should not be used to treat low blood glucose?
  - 3 hard candies
  - 1/2 cup orange juice
  - 1 cup diet soft drink
  - 1 cup skim milk
9. For a person in good control, what effect does exercise have on blood glucose
  - Lowers it
  - Raises it
  - Has no effect
10. What effect will an infection most likely have on blood glucose?
  - Lowers it
  - Raises it
  - Has no effect
11. The best way to take care of your feet is to:
  - Look at and wash them each day
  - Massages them with alcohol each day
  - Soak them for one hour each day
  - Buy shoes a size larger than usual
12. Eating foods lower in fat decreases your risk for:
  - Nerve disease
  - Kidney disease
  - Heart disease
  - Eye disease
13. Numbness and tingling may be symptoms of:
  - Kidney disease
  - Nerve disease
  - Eye disease
  - Liver disease
14. Which of the following is usually not associated with diabetes:
  - Vision problems
  - Kidney problems
  - Nerve problems
  - Lung problems.

The original instrument was well-developed, validated, and widely used to assess general diabetes knowledge. It is a simple and user-friendly instrument and it has been translated into many languages [10]. It has consistently achieved acceptable results in Cronbach's alpha-validity tests [7]. Further, the results of this study are consistent with AlJohani et al. (2016) who used similar methods for the translation and validation of the summary of diabetes self-care activities survey into Arabic [17].

In conclusion, this Arabic version of DKT-2 has been demonstrated to be a reliable and valid measure of diabetes knowledge which can be used in clinical practice. However, the sample sizes used by this study for testing-retesting, as well as the convenience sampling methods, may not be representative of all situations in Saudi Arabia.

## Figures and Tables

**Table 1:** Expert panel characteristics.

ID	Gender	Profession	Work in healthcare	Diabetes experience (years)
1	F	Head nurse	Yes	3
2	F	Staff nurse	Yes	8
3	F	Head of diabetes education	Yes	6
4	M	Nurse manager	Yes	14
5	M	Staff nurse 1	Yes	10
6	F	Nursing student	No	0
7	M	Clinical-research consultant	Yes	22
8	F	Nurse	Yes	1.5
9	M	Translator	Yes	0

**Table 2:** Demographics.

Characteristics	Frequency (*n*)	Percent %
*HbA1c level*		
Good control	28	20.1
Acceptable control	39	28.1
Poor control	71	51.1
Missing	1	0.7

*Age group*		
18–30 years	56	40.3
3–45 years	34	24.5
46–55 years	20	14.4
>56 years	25	18
Missing	4	2.9

*Gender*		
Male	62	44.6
Female	74	53.2
Missing	3	2.2

*Monthly income*		
<5.000	56	40.3
<10.000	44	31.7
<15.000	14	10.1
>16.000	19	13.7
Missing	6	4.3

*Smoking*		
Currently	16	11.5
No	95	68.3
Yes	14	10.1
Missing	14	10.1

*Diagnosis time*		
<2 years	7	5.0
2–4 years	18	12.9
5–7 years	27	19.4
8–10 years	21	15.1
>10 years	61	43.9
Missing	5	3.6

*Education level*		
Elementary-school	16	11.5
Middle-school	14	10.1
High-school	45	32.4
Bachelor	57	41.0
Postgraduate	4	2.9
Missing	3	2.2

*Other diseases*		
Cardiac	7	5.0
Bp	13	9.4
Kidney	3	2.0
Eye	20	14.4
>1	53	38.1
Missing	43	30.9

**Table 3:** Intraclass correlation coefficient (ICC).

	Intraclass correlation^b^	95% confidence interval		*F* test with true value 0			
		Lower-bound	Upper-bound	Value	df1	df2	Sign
Single measures	0.822^a^	0.573	0.920	13.418	33	33	0.000
Average measures	0.903^c^	0.729	0.958	13.418	33	33	0.000

*Note*. Two-way mixed-effects model where people effects are random and measures effects are fixed.

^a^The estimator is the same whether the interaction effect is present or not.

^b^Type A ICCs using an absolute agreement definition.

^c^To achieve an estimate, this number is computed assuming the interaction effect is absent.

**Table 4:** Content validity index (CVI).

Item description	Expert						Number of agreements	I-CVI
	1	2	3	4	5	6		
Scale item 1	3	3	4	3	4	4	6	1
Scale item 2	3	3	3	4	3	4	6	1
Scale item 3	4	3	4	3	3	3	6	1
Scale item 4	3	3	3	4	3	2	5	0.833
Scale item 5	4	3	2	4	4	4	5	0.833
Scale item 6	4	4	3	3	4	4	6	1
Scale item 7	4	3	4	3	4	4	6	1
Scale item 8	4	4	4	4	4	3	6	1
Scale item 9	3	4	3	3	2	3	5	0.833
Scale item 10	3	4	3	3	4	4	6	1
Scale item 11	4	3	4	3	4	3	6	1
Scale item 12	3	3	3	4	3	4	6	1
Scale item 13	3	4	3	3	4	3	6	1
Scale item 14	4	4	4	3	4	4	6	1
							S-CVI/Ave	**0.964**
							Total agreement	**11**
							S-CVI/UA	**0.785**
